# Aluminum for Dental Purposes

**Published:** 1870-10

**Authors:** J. U. L. Feemster


### ALUMINUM FOR DENTAL PURPOSES.

BY J. U. L. FEEMSTER, D. D. S.

Aluminum is endowed with properties which peculiarly adapt it to the use of supplying artificial dentures. The properties that present themselves so prominently are lightness, strength and resistance to chemical agents. Its specific gravity being when pure and cast only 2.54 to 2.55, occupying almost four and one half-times the space of an equal weight of silver, and about eight times as much as gold, and very near ten times that of platinum. Its strength when compared to silver, gold or platinum is about equal to these well known precious metals. The indifference with which it comports itself to all the chemical agents that it is likely to come in contact with when in use as a base for artificial teeth is a very great consideration in its favor. When once in the mouth it is coated with a very thin film of corundum, which effectually resists further oxidation and even resists the action of nascent chlorine. A further consideration that renders it preferable to all other known elements or compounds is that when in combination its compounds are harmless to the animal economy. In addition to all these properties and considerations, it is a great conductor of caloric, and permits the ingress and egress of this agent to the mucous membrane and subjacent tissues covered by artificial dentures, cooling and refreshing the parts when cool drinks are taken, and warms and soothes when warm beverages are used. These considerations or changes are necessary in the divine adaptation and fitness of things, to maintain these parts in a normal condition. All admit that these properties of aluminum make it the most desirable element known for the use of the Dentist, and no known combination of elements is so well adapted to this purpose; but some of its properties that are so desirable when in use become great difficulties in preparing it for use. Its lightness has heretofore rendered it very difficult to cast into proper shape, and its indifference to chemical agents rendered it next to impossible to solder it as it should be, and if soldered the alloy with which it was united rendered it susceptible of being corroded more readily, and often the compound resulting from the corrosion of the alloy was deleterious to vitality. But when aluminum is subjected to the *elastic* force and *deoxidizing* influence of hydrogen, these difficulties, which seemed to be solid angularities of fact, are dissipated into shining ether, and a solid, thin, perfect fitting and shining aluminum plate easily produced, *cast* directly to the teeth, thus dispensing with, not only the perplexity of soldering, but with all the unpleasant results of corodible and deleterious alloys. Then the shrinkage of the aluminum while congealing and the contraction while cooling from the congealing point to the common temperature of the atmosphere, presented a very difficult technical problem to be solved. Here, apparently, insurmountable difficulties have also been overcome, and now the teeth we used to attach to vulcanite base can be imbedded in a flask and an aluminum plate cast directly on the teeth, and neither the artificial gums or teeth injured. The necessary conditions of success are, to have hydrogen under pressure and such an arrangement that the elastic force can be made to act on the fused aluminum and inject it into the angles of the mould, and to prevent the metal from lapping over the edges of the gums, so that the central contraction will break them in case the metal is permitted to flow over and outside, as has often been done. Then the shrinkage in congealing is compensated for or prevented by supplying a reversion of molten aluminum for the congealing plate to draw from. The aluminum plate is easily polished by grinding to an even surface with English corundum wheels and cones and working in caustic potassa solution, and then in water, drying with a clean napkin, and using parafine on the burnisher by heating the burnisher hot enough to melt the parafine, and applying it to the parafine as often as necessary, to keep the burnisher working smoothly. The best fits I ever made or ever saw were made by casting aluminum in this way. Here cases of excellent fits are not casual or occasional, but almost universal, a misfit being the exception. The expansion of the plaster cast is exactly compensated by the contraction of the aluminum.

